# Core decompression combined with platelet-rich plasma-augmented bone grafting for femur head necrosis: a systematic review and meta-analysis

**DOI:** 10.1097/JS9.0000000000001028

**Published:** 2024-01-04

**Authors:** Bo Zhu, Jianmin Li, Xuejia Li, Shengyi Feng, Bo Li

**Affiliations:** Department of Orthopedics, Yueyang Hospital of Integrated Traditional Chinese and Western Medicine, Shanghai University of Traditional Chinese Medicine, Shanghai, People’s Republic of China

**Keywords:** core decompression and bone grafting, femur head necrosis, meta-analysis, platelet-rich plasma, systematic review

## Abstract

**Background:**

The clinical potential of biologic augmentation in core decompression and bone grafting for femoral head necrosis is widely acknowledged, with platelet-rich plasma (PRP) being a frequently employed biologic adjunct. However, its clinical application is not standardized, and high-level evidence is lacking. This study aimed to evaluate the efficacy and safety of core decompression and bone grafting combined with PRP for femur head necrosis.

**Methods:**

Several databases were systematically retrieved for randomized controlled trials comparing core decompression and bone grafting combined with or without PRP. A systematic review and meta-analysis were conducted following the PRISMA 2020 and AMSTAR 2 guidelines. The study is registered with PROSPERO under the code CRD42022361007, and it is also listed in the research registry under the identification number reviewregistry1537.

**Results:**

Eleven studies with 642 participants (742 hips) were included. The pooled estimates revealed that when core decompression and bone grafting were combined with PRP, the Harris hip score (mean difference: 7.98; 95% CI: 5.77–10.20; *P*<0.001), visual analog scale (SMD: −0.68; 95% CI: −0.96 – −0.40; *P*<0.001) and the pain component of Harris hip score (SMD: 8.4; 95% CI: 4.12–12.68; *P*<0.001), and reduction of radiographic progression [risk ratio (RR): 0.40; 95% CI: 0.27–0.59; *P*<0.001] were superior to core decompression and bone grafting alone. Fewer patients with treatment failure (RR: 0.27; 95% CI: 0.14–0.52; *P*<0.001) and higher good-to-excellent results (RR: 1.48; 95% CI: 1.17–1.86; *P*<0.001) were observed in treatment groups than control groups. Meanwhile, the pooled analysis substantiated the superior safety profile of PRP (RR: 0.29; 95% CI: 0.11–0.77; *P*=0.01).

**Conclusions:**

The combination of core decompression and bone grafting with PRP is superior to the approach without PRP, demonstrating enhanced effectiveness in terms of function, pain relief, and radiographic progression. Additionally, it results in lower rates of treatment failure and adverse events. However, further high-quality RCTs are needed to evaluate their effectiveness due to methodological and implementation limitations observed in the existing evidence.

## Introduction

Femur head necrosis, also called osteonecrosis of the femoral head (ONFH), refers to subchondral osteonecrosis and collapse caused by abnormal microcirculation of the femoral head and causes progressive and secondary arthritis^1^, which is more common in men aged 30–50 years^2^. Corticosteroid use, excessive alcohol consumption, and trauma are the most common causes^3^. It is estimated that there are more than 20 million patients with femur head necrosis worldwide, and there are about 8.12 million patients in China, with an annual growth rate of 100 000–200 000^4^. In the United States, there are at least 300 000–600 000 patients with ONFH^5,6^, with an annual growth rate of 10 000–20 000^3,4,6^.

ONFH often causes severe pain and degenerative joint disease. If not treated in time, more than 85% of patients develop subchondral bone plate collapse within 2 years, which can progress to femoral head collapse and hip osteoarthritis^2,7^ and eventually require total hip arthroplasty (THA)^7,8^. Given the young age of ONFH patients, the risk and probability of surgical revision and related complications are significantly high^1^. Moreover, nonoperative treatment modalities are generally ineffective for halting progression^3,8,9^. Accordingly, except for small-sized, medially located lesions (<10%), joint-preserving procedures should be attempted in early-stage lesions to save the femoral head^8^. An increasing body of evidence suggests that core decompression, one of the earliest hip-preserving procedures, exhibits superior efficacy than the nonsurgical treatment of precollapse lesions^8–10^. However, the therapeutic effect of core decompression remains subject to significant controversy^11^, and its long-term efficacy lacks clinical evidence based on large sample size-based studies^1,8,12^. A meta-analysis of 32 studies involving 2441 hips with an average follow-up of 54.3 months reported an overall success rate of only 65%^13^.

At present, the use of single-hole intramedullary decompression alone is limited in clinical practice, mainly due to the reduction of biomechanical strength and stress concentration in the femoral head after decompression and removal of dead bone, which increases the risk of collapse and fracture of the femoral head^5,14^. Additionally, core decompression did not yield superior effects in promoting bone regeneration in necrotic areas^15,16^. Importantly, adjunctive PRP-based augmentation in CD and bone grafting has yielded promising results and has gradually become a research hotspot^17^.

PRP is a blood product obtained by centrifugation of autologous peripheral blood with a higher platelet concentration than baseline, which contains a variety of growth factors and plays an important role in tissue repair and the proliferation and differentiation of mesenchymal stem cells^18,19^. PRP combined with bone grafting enhances the mechanical strength of the femoral head and the ability of bone tissue regeneration^19^. In recent years, much emphasis has been placed on its clinical application in ONFH^18,20^, but high-level evidence is still lacking. Herein, we assessed the clinical efficacy of core decompression and bone grafting with or without PRP for treating ONFH. Furthermore, we provided a comprehensive overview of the potential mechanism by which PRP enhances the therapeutic effects of ONFH, drawing upon existing findings related to PRP’s impact on tissue repair.

## Methods

This review was reported in line with the Preferred Reporting Items for Systematic Reviews and Meta-Analysis (PRISMA) 2020 statement^21^ (Appendix 1, Supplemental Digital Content 1, http://links.lww.com/JS9/B620) and has been registered in PROSPERO (registration ID: CRD42022361007) and Research Registry (UIN: reviewregistry1537, https://www.researchregistry.com/). The methodological quality of this systematic review was evaluated to be of high-quality using the Assessing the Methodological quality of Systematic Reviews (AMSTAR) 2 checklist (Appendix 2, Supplemental Digital Content 1, http://links.lww.com/JS9/B620)^22^.

### Search strategy

We searched PubMed, Embase, Cochrane Central Register of Controlled Trials (CENTRAL), China Network Knowledge Infrastructure (CNKI), China Science and Technology Journal Database (CSTJ), Wan Fang Database, and Chinese Biomedicine (CBM) databases from their inception dates until 30 September 2022, for studies that determined the efficacy and safety of core decompression and bone grafting combined with PRP for femur head necrosis. We combined keywords from MeSH headings with self-generated keywords to identify studies in English and Chinese. The search strategy was: (((femur head necroses) OR (osteonecrosis of the femoral head) OR (necroses) OR (osteonecrosis)) AND ((platelet-rich plasma) OR (Platelet-Rich Fibrin) OR (PRP)) AND ((bone grafting) OR (graft) OR (Bone Transplantation) OR (decompression) OR (core decompression))) AND ((“1900/1/1”[Date - Publication]: “2022/9/30”[Date - Publication])).

### Inclusion and exclusion criteria

RCTs were included if they met the following inclusion criteria: (1) studies with explicit diagnostic standards and meeting the criteria for femur head necrosis; (2) included patients were affected by Association Research Circulation Osseous (ARCO) staging^23^ or Ficat and Arlet (Ficat) classification^24^, grades 1–3, regardless of race, sex, and age; (3) the treatment group was treated with core decompression and bone grafting combined with PRP, and the control group was treated with core decompression and bone grafting only, regardless of nonvascularized/ vascularized bone grafting and synthetic bone substitutes; (4) studies that reported at least one outcome of interest. Exclusion criteria for this review included studies employing stem cell augmentation or other biological adjuncts, studies unrelated to the theme or lacking complete data, as well as letters, cases, reviews, and conference abstracts.

### Outcomes

The primary outcomes used in this review were functional outcome measure [assessed with Harris hip score (HHS)], pain [mainly assessed by visual analog scale (VAS)], and radiographic progression (including progression of disease stage, new collapses, and aggravation of necrosis judged by the authors with imaging). Given that various classification systems were applied and clinical progression (including collapse) is different from the radiographic progression, both reflect disease progression, and they were stratified into different groups during subgroup analysis. Moreover, studies were excluded if the description of imaging changes was vague, including ‘no obvious bone ingrowth’, ‘no double-line sign’, or ‘high signal in the head’, etc. Secondary outcomes were treatment failure (defined by further surgery for femoral head collapse, disease progression, and subjective assessment of symptom exacerbation), good-to-excellent results (HHS ≥80)^25^, and major adverse events (infection around the implant, thrombosis, rejection, immune reaction, allergic reaction, or any other adverse effect that caused the patient to leave the trial through medical advice or willingly).

### Extraction criteria

All included studies were independently screened by two researchers (Bo Zhu and Shengyi Feng). Standardized data extraction tables were used to extract data, including leading author, publication year, age, course, disease stage, randomization, sample size, interventions (treatment group and control group), outcome criteria, adverse reactions, and follow-up time. After contacting authors and journal publishers for verification, we excluded duplicates, studies with incomplete data, and studies for which the full text was not retrieved. Outcome data were also excluded if reported as graphs without exact values or in other unusable forms. The Cohen’s Kappa score was calculated to show agreement between the reviewers. Any disagreements regarding study selection or data extraction were resolved by discussion with a third author when necessary (Bo Li).

### Risk of bias and quality of evidence

Two researchers (Bo Zhu and Shengyi Feng) independently assessed the risk of bias using The Cochrane Risk of Bias Tool^26^ and Review Manager 5.3 software (The Cochrane Collaboration). Selection bias (random sequence generation and allocation concealment), performance bias (blinding of participants and personnel), detection bias (outcome assessment blinding), attrition bias (incomplete outcome data), reporting bias (selective reporting), and other bias (such as major baseline imbalance and undue influence from funders) were assessed. A total of seven domains were evaluated as low risk of bias, high risk of bias, and unclear risk of bias. In case of disagreements, a consensus was reached after a discussion. The quality of evidence provided by the pooled results was assessed according to the Grading of Recommendations Assessment, Development, and Evaluation (GRADE) guidelines provided by GRADEpro (https://www.gradepro.org/)^27^.

### Statistical analysis

Review Manager 5.3 was utilized to assess all statistical analyses, and *P*-values <0.05 were statistically significant. Dichotomous outcomes were expressed as risk ratios (RRs), and continuous outcomes were presented as mean difference (MD) and standard mean difference (SMD), with 95% CI. Heterogeneity across studies was assessed by visual analysis of forest plots, and the degree of heterogeneity was measured by *I* ^2^. When heterogeneity was not significant (*I* ^2^<50%), a fixed-effect model was used. Otherwise, a random-effects model was used since it provides a more conservative effect size estimate. In case of considerable heterogeneity, further descriptive analysis or subgroup analysis was performed, and the stability of the results was verified by sensitivity analysis. In addition, if the number of trials reporting the same outcome was ≥10, a funnel plot was used to analyze potential publication bias.

## Results

### Included studies and the characteristics

**Figure 1:**
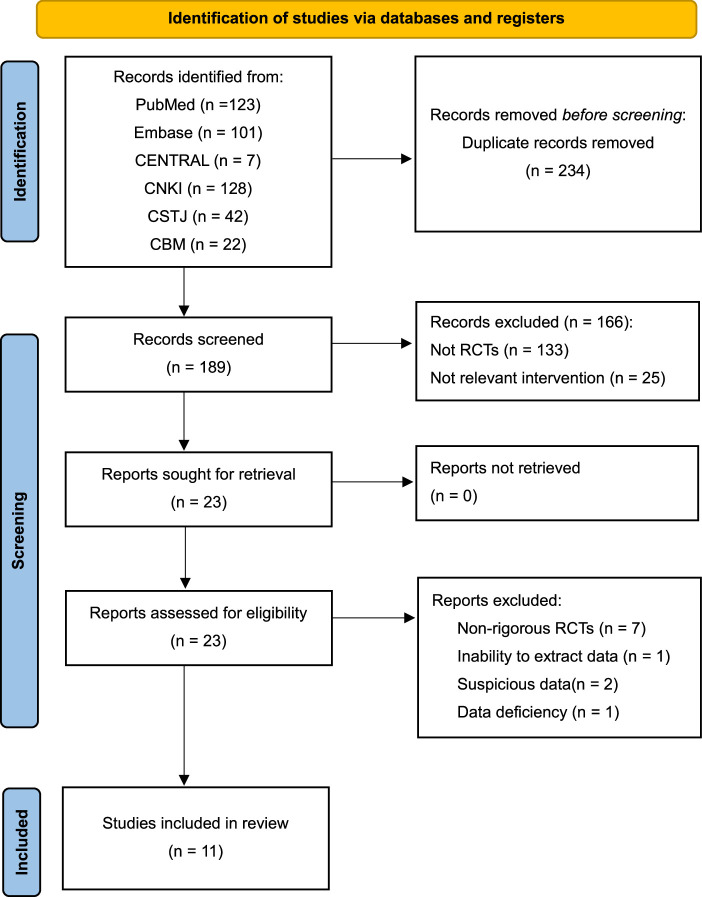
PRISMA 2020 flow diagram Flow diagram depicting the screening process.

The initial literature search yielded 423 citations, and 234 duplicate articles were excluded. One hundred eighty-nine articles were further excluded after screening the titles and abstracts. After reading the full text of the remaining 23 articles, 11 trials that met the inclusion criteria were finally included^20,28–37^(Fig. 1). The Cohen’s Kappa value was 0.913, implying a high-level of agreement between the reviewers (Appendix 3, Supplemental Digital Content 1, http://links.lww.com/JS9/B620).

A total of 642 patients (742 hips) with femur head necrosis were included in the 11 studies, with ARCO or Ficat stages I–III, followed up from 6 to 72 months. RCTs were published in Chinese and English, with 10^28–37^ originating from China and one^20^ from India. All studies documented the diagnostic criteria by detailing or citing references. In two studies, 7^35^ and 46^37^ patients underwent unilateral post-traumatic femur head necrosis, while the remaining studies analyzed nontraumatic cases, including alcoholic, steroid-associated, and idiopathic types. Nonvascularized bone grafting was used in all studies, which involved curetting the necrotic area and decompression, followed by cavity filling with bone grafts such as autografts, allografts, and bone graft substitutes. Six studies used autologous bone (five used iliac bone^31,33,35–37^, one used fibula^20^), one study used allogeneic fibula^30^, and four studies used artificial bone^28,29,32,34^. Bone grafting in eight studies^20,28,29,31,33,34,36,37^ was performed through the core decompression channel (Phemister technique) or with a small window through the head-neck junction in three studies^30,32,35^ (light bulb procedure).

For PRP preparation, blood samples were centrifuged by double spin in nine studies^28–36^ and single spin in two studies^20,37^, of which one^20^ used a leukocyte filter. Three studies^30,35,37^ provided the relative centrifugal force (RCF), and the remaining eight^20,28,29,31–34,36^ provided data including rotation speed (rpm) and/or centrifugal radius (R), which were converted to RCF using the formula^38^: RCF(g)=1.119×10^-5^×R×(rpm)^2^.

Regarding outcomes, HHS was used as one of the primary outcomes in all studies; one study^37^ assessed the minimum clinically important difference (a 10-point increase in the HHS), and five studies^20,28,30,32,33^ reported good-to-excellent results (HHS ≥80)^27^. Two studies^28,30^ which used HHS ≥75 to grade good-to-excellent results, were excluded. VAS and the pain component of HHS were used in nine studies^28–35,37^ and one study^20^ to assess pain, respectively. Radiographic progression was reported in 10 studies^20,28–34,36,37^. In this respect, five studies^20,28–30,34^ (all patients were precollapse cases) reported the number of new collapses, two studies^33,36^ reported staging progression (one^36^ was excluded because data were not available), and four studies^20,31,32,37^ reported radiographic progression, of which two^31,37^ used radiological failure criteria (defined as the occurrence of new collapses or the occurrence of increased collapses greater than 2 mm^39^), and the remaining two^20,32^ were interpreted based on the imaging findings (one^32^ was excluded for a vague description of radiographic progression). Seven studies^20,28,29,32–34,37^ reported reoperation (including THA and transtrochanteric rotational osteotomy), and six studies^20,28,29,34–36^ reported adverse reactions. The basic characteristics of the included studies are presented in Table 1.

**Table 1:** Characteristics of included trials.

	Sample (patient/hip, age, course, staging)		Interventions		PRP preparation							
Study	T	C	T	C	Spin	Duration (min)	rpm (r)	RCF (g)	Activator	Bone grafting (materials, procedure)	Follow-up	Outcomes
Chai *et al*.^28^	29/29 43.73±3.25y NR ARCO Ⅱ	25/25 44.33±3.17y NR ARCO Ⅱ	CB +PRP	CB	Double	20, 10	1500	—	NR	β-TCP Phemister	12 m	HHS, VAS, RP, TF, AE
Niu and Wang^31^	36/36 37.58±9.24y NR ARCO Ⅰ-Ⅱ	36/36 37.24±9.81y NR ARCO Ⅰ-Ⅱ	CB +PRP	CB	Double	10, 10	1500	—	NR	autograft Phemister	6 m	HHS, VAS, RP
Zhang^35^	41/41 37.58±10.26y 17.98±9.26 m ARCO Ⅱ-Ⅲ	40/40 38.72±11.37y 19.13±8.75 m ARCO Ⅱ-Ⅲ	CB +PRP	CB	Double	20, 10	NR	1500	NR	autograft light bulb	6 m	HHS, VAS, AE
Aggarwal *et al*.^20^	19/25 38.2±10.4y NR Ficat Ⅰ-Ⅱ	21/28 35.2±12.5y NR Ficat Ⅰ-Ⅱ	CB +PRP	CB	Single	15	1500	—	CaCl^2^	autograft Phemister	54 m–72 m	HHS, RP, TF, GTER, PCOH
Chen *et al*.^29^	50/80 43.47±7.23y 15.8±2.9 m ARCO Ⅱ	50/80 45.72±7.43y 14.9±3.8 m ARCO Ⅱ	CB +PRP	CB	Double	20, 10	1500	—	NR	β-TCP Phemister	12 m	HHS, VAS, RP, TF, AE
Yuan *et al*.^34^	19/19 45±11y NR Ficat Ⅰ-Ⅱ	20/20 41±14y NR Ficat Ⅰ-Ⅱ	CB +PRP	CB	Double	15, 20	3500	—	NR	β-TCP Phemister	18 m	HHS, VAS, RP, TF, AE
Wang *et al*.^32^	34/34 39.29±6.67y 14.76±4.67 m ARCO Ⅰ-Ⅱ	31/31 37.16±7.16y 13.83±5.51 m ARCO Ⅰ-Ⅱ	CB +PRP	CB	Double	10, 10	2000, 2200	671.4, 812.4	NR	β-TCP light bulb	12 m	HHS, VAS, RP, TF
Xian *et al*.^37^	24/24 28.3±1.4y 15.7±0.7 m ARCO Ⅱ-Ⅲ	22/22 29.6±1.7y 17.1±0.8 m ARCO Ⅱ-Ⅲ	CB +PRP	CB	Single	8	NR	500	CaCl^2^	autograft Phemister	36–60 m	HHS, VAS, RP, TF, GTER
Jiang *et al*.^30^	26/35 18-65y 3-6 m ARCO Ⅱ	24/32 20-63y 3-6 m ARCO Ⅱ	CB +PRP	CB	Double	20, 10	NR	1500	NR	allograft light bulb	6 m	HHS, VAS, RP
Zhao *et al*.^36^	30/32 40.21±5.12y 15.02±3.44 m Ficat Ⅰ-Ⅲ	30/33 39.25±6.01y 14.35 ±3.48 m Ficat Ⅰ-Ⅲ	CB +PRP	CB	Double	10, 10	2000	671.4	NR	autograft Phemister	12 m	HHS, RP, AE
Yang *et al*.^33^	15/20 35.6±2.4y NR Ficat Ⅰ-Ⅱ	20/20 37.2±7.1y NR Ficat Ⅰ-Ⅱ	CB +PRP	CB	Double	10, 10	2000	671.4	NR	autograft Phemister	12 m	HHS, VAS, RP, TF, GTER

AE, adverse events; CB, Core decompression and bone grafting; GTER, good-to-excellent results; NR, not reported; PCOH, pain component of HHS; R, radius; RCF, relative centrifugal force; RP, radiographic progression; rpm, Rotation Speed; TF, treatment failure; β-TCP, β-Tricalcium phosphate.

In addition, the descriptions of postoperative management were not specific enough and varied greatly to classify and discuss, and the quality of implementation is difficult to determine. Therefore, it is reported in supplementary documents (Appendix 4, Supplemental Digital Content 1, http://links.lww.com/JS9/B620).

### Risk of bias

**Figure 2:**
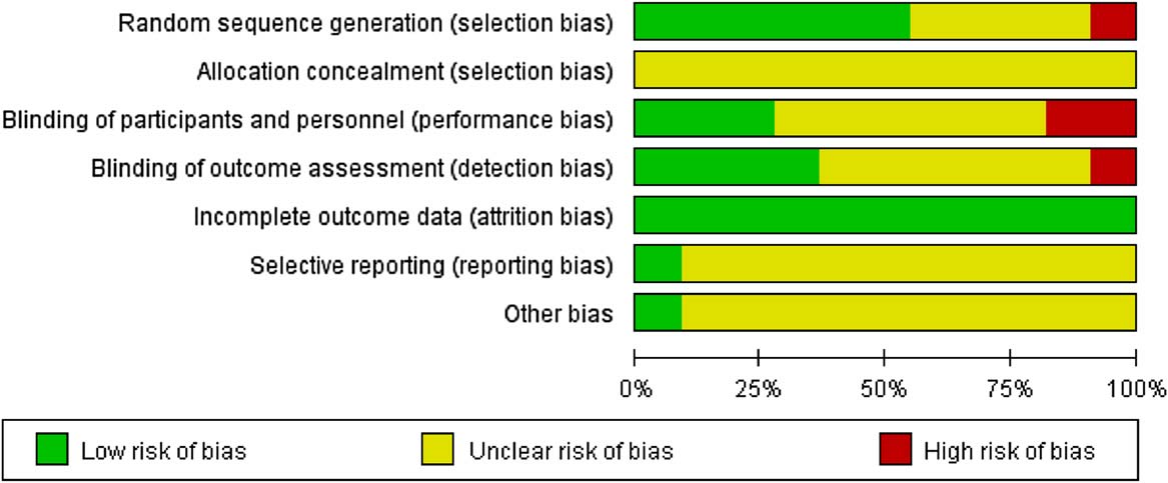
Risk of bias assessment graph.

Seven trials reported the method of random sequence generation involving random number tables (*n*=4)^28,33–35^, computer-generated algorithms (*n*=2)^20,32^, and sealed envelopes (*n*=1)^37^. One trial^31^ used the ‘principle of balanced and comparable basic characteristics’ for semi-randomized grouping, while three studies^29,30,36^ did not mention the details of the random sequence. The blinding method mentioned in five studies was double-blinded (the participants and the assessor, *n*=2)^20,34^, or single-blinded (*n*=3)^28,33,37^. Allocation concealment was unclear in all the included studies. Loss to follow-up and dropout were reported in seven studies^20,28–30,32,33,37^. In one study^32^, patient dropouts were due to a car accident (*n*=1) and fall (*n*=1). In another study^37^, four patients were lost to follow-up for unreported causes, while all patients completed the follow-up in five studies^20,28–30,33^. The risk of publication bias was unclear for all studies (Fig. 2). In addition, the surgical technique (light bulb procedure or Phemister technique), disease stage (precollapse or postcollapse), source of the bone graft (autogenous, allogeneic, or artificial), type of postoperative management (load limit, medicine, and rehabilitation exercise) and differences in PRP preparation represented potential sources of bias.

### Primary outcomes

#### Function (HHS)

**Figure 3:**
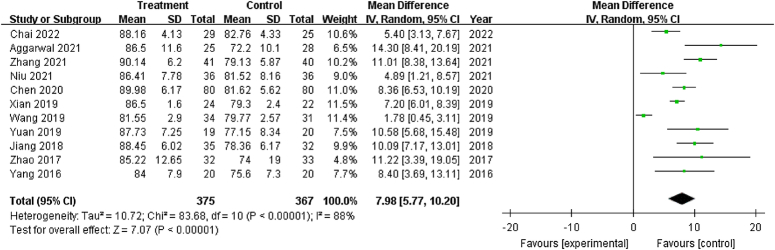
Forest plot of the Harris Hip Score of patients that underwent core decompression and bone grafting with and without platelet-rich plasma.

The HHS was evaluated in all included trials. Given that high heterogeneity was present (χ^2^: 83.68; *P*<0.001; *I* ^2^=88%), a random-effects model was used (Fig. 3). The pooled results indicated that core decompression combined with PRP-augmented bone grafting was superior to the same surgery without PRP (MD: 7.98; 95% CI: 5.77–10.20; *P*<0.001).

**Figure 4:**
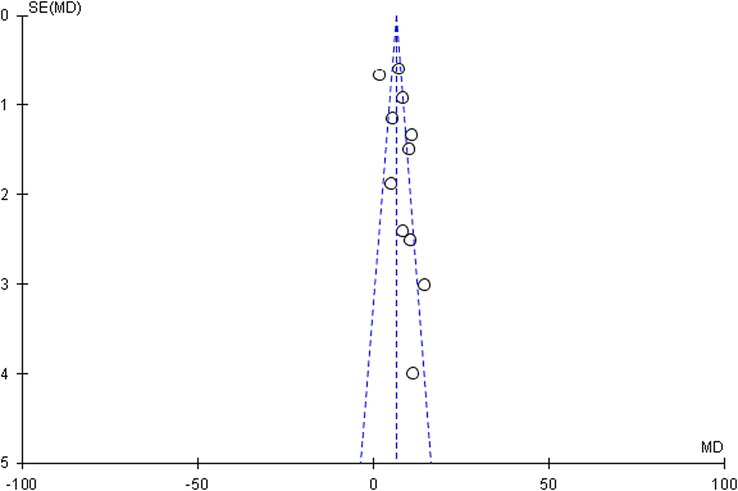
Funnel plot of the Harris Hip Score of patients that underwent core decompression and bone grafting with and without platelet-rich plasma.

A sensitivity analysis was subsequently performed to identify sources of heterogeneity. Significant heterogeneity (*I* ^2^ above 87%) was observed after excluding any study, except for one study^32^ (*I* ^2^=60%). After substratification into ‘windowing of the femur head-neck junction’, ‘artificial bioceramic bone’, and ‘secondary centrifugation for PRP preparation’, significant heterogeneity remained. We speculate that considerable heterogeneity may be attributed to the subjective description of pain when filling out the HHS scale, and it should be borne in mind that different analgesics were prescribed to these patients. Since more than 10 studies evaluated the HHS, a funnel plot was generated to determine publication bias. The funnel plot (Fig. 4) exhibited an asymmetric distribution, suggesting the presence of publication bias.

#### Pain (VAS and pain component of HHS)

**Figure 5:**
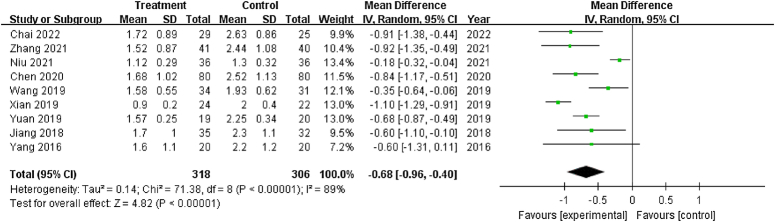
Forest plot of the visual analog scale of patients that underwent core decompression and bone grafting with and without platelet-rich plasma.

VAS and the pain component of HHS were used in nine studies^28–35,37^ and one study^20^, respectively, to assess pain. Given that significant heterogeneity was observed (χ^2^: 71.38; *P*<0.001; *I* ^2^= 89%), a random-effects model was used (Fig. 5). The pooled estimates favored core decompression combined with PRP-augmented bone grafting compared with the same surgery without PRP (SMD: −0.68; 95% CI: −0.96 – −0.40; *P*<0.001). Sensitivity analysis showed that the heterogeneity was significantly reduced after excluding two studies^31,37^ (χ^2^: 8.25; *P*=0.22; *I* ^2^=27%). Significant selection and performance biases were observed in both studies; one^31^ was quasi-randomized, and all patients included in the other^37^ were post-traumatic (many with polytrauma at the time of initial injury), and PRP was prepared by single centrifugation.

The pain component of HHS in one study^20^ was significantly improved in the treatment group (SMD: 8.4; 95% CI: 4.12–12.68; *P*<0.001) compared with the control group. A higher score of the pain component of HHS represented better pain relief.

#### Radiographic progression

After excluding two studies^32,36^ with inadequate data, 8 out of 10 studies that reported radiographic progression were finally included^20,28–31,33,34,37^. In six studies, stratification was based on the incidence of collapse; one^33^ reported the number of patients who progressed from Ficat I–II (precollapse) to Ficat Ⅲ (postcollapse), and five^20,28–30,34^ (all patients were precollapse cases) reported the number of new collapses. Three studies reported radiographic progression, among which two^31,37^ used radiological failure criteria^39^, and one^20^ was based on imaging findings and assigned collapse and radiographic progression to two different subgroups.

**Figure 6:**
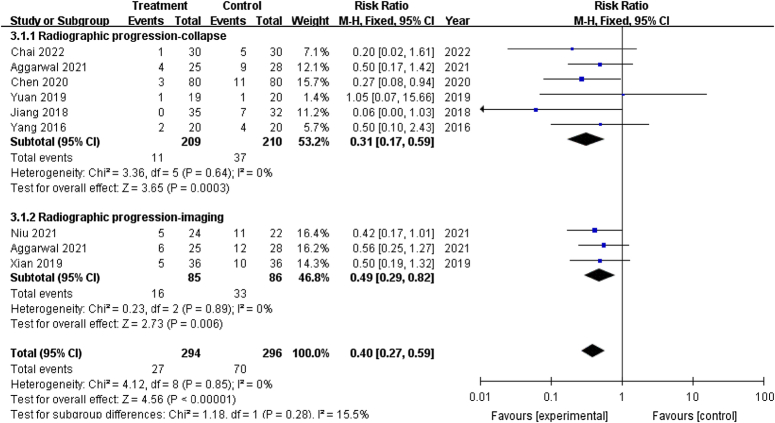
Forest plot of radiographic progression in patients that underwent core decompression and bone grafting with and without platelet-rich plasma.

The pooled estimates showed that the femoral head collapse rate (RR: 0.31; 95% CI: 0.17–0.59; *P*<0.001), radiographic progression (RR: 0.49; 95% CI: 0.29–0.82; *P*=0.006) and the overall effect(RR: 0.40; 95% CI: 0.27–0.59; *P*<0.001) in treatment groups was significantly lower than control groups (Fig. 6). Furthermore, no heterogeneity in both subgroups was found.

### Secondary outcomes

#### Treatment failure

**Figure 7:**
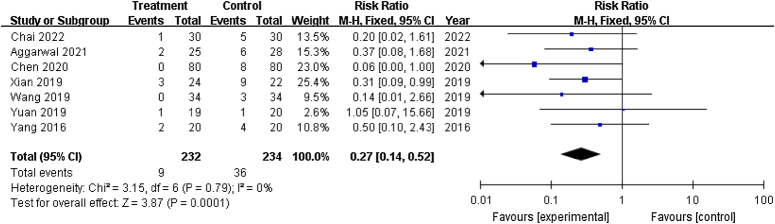
Forest plot for treatment failure of patients that underwent core decompression and bone grafting with and without platelet-rich plasma.

Seven studies^20,28,29,32–34,37^ reported reoperation (including THA and transtrochanteric rotational osteotomy). The pooled results showed a significantly lower number of patients with treatment failure in treatment groups than in control groups (RR: 0.27; 95% CI: 0.14–0.52; *P*<0.001) (Fig. 7).

#### Good-to-excellent results

**Figure 8:**
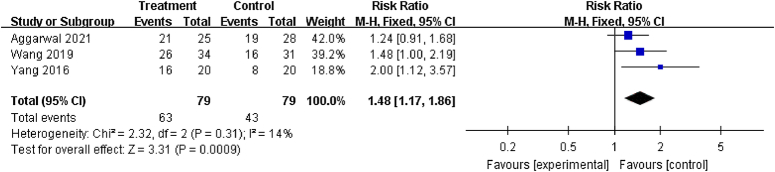
Forest plot of good-to-excellent results for patients that underwent core decompression and bone grafting with and without platelet-rich plasma.

Three studies^20,32,33^ reported good-to-excellent results (HHS ≥80). Compared with control groups, treatment groups were associated with significantly higher rates of good-to-excellent results at final follow-up (RR: 1.48; 95% CI: 1.17–1.86; *P*<0.001) (Fig. 8).

#### Adverse events

**Figure 9:**
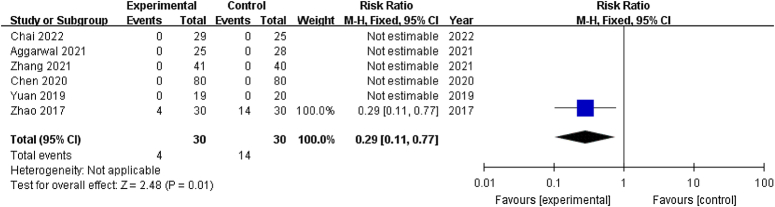
Forest plot of adverse events for patients that underwent core decompression and bone grafting with and without platelet-rich plasma. (RR: 0.29; 95% CI: 0.11–0.77; *P*=0.01).

Five studies^20,28,29,34,35^ reported no complications during follow-up, and one^36^ reported 4 and 14 complications in the treatment group and the control group, respectively, including redness and swelling, postoperative infection, hypovolemic shock, and deep vein thrombosis of the lower extremities. The pooled analysis of adverse events substantiated the superior safety profile of PRP (RR: 0.29; 95% CI: 0.11–0.77; *P*=0.01). The lack of strict criteria for related complications might have introduced subjectivity into the analysis. Researchers might overlook minor complications, making it challenging to infer the absence of adverse events in other studies (Fig. 9).

#### Evidence evaluation

The GRADE tool was used to assess the quality of evidence. The level of evidence was deemed very low for the HHS and VAS, low for good-to-excellent results, while it reached a moderate level for radiographic progression and treatment failure (Appendix 5, Supplemental Digital Content 1, http://links.lww.com/JS9/B620).

## Discussion

Our pooled estimates showed that compared with core decompression and bone grafting alone, the combination of PRP improved joint function (HHS, *P*<0.001), reduced pain (VAS, *P*<0.001; pain component of HHS, *P*<0.001), delayed radiographic progression (*P*<0.001) and obtained a fewer treatment failure (*P*<0.001) and higher good-to-excellent results (*P*<0.001) to a certain extent. Meanwhile, our findings showed that hip-preserving surgery with PRP was safe (adverse events, *P*=0.01), given that five out of six studies that mentioned complications reported no cases of localized complications postoperatively, and one study^36^ documented fewer adverse events in the treatment group compared to the control group (4/30 vs. 14/30). Inflammation and infection were the main adverse events, indicating that the intraoperative or postoperative procedures were not conducted after strict aseptic conditions. Although PRP exhibits significant antibacterial activity when applied during traumatic bone infection^40^, the presence of detection bias cannot be excluded from the treatment group. It is well-established that PRP is derived from autologous blood without immune rejection, and its safety has been confirmed by clinical studies^19^. Nevertheless, the residual red blood cells in PRP may disintegrate, cause hemolysis, and promote inflammation^41^. Moreover, proinflammatory substances released by the residual leukocytes cause tissue damage^42^. However, it is widely believed that leukocytes in PRP do not increase the risk of adverse reactions^43^, instead inducing significantly higher proliferation of bone marrow mesenchymal stem cells^44^.

The integration of CD with bone graft therapy enhances the mechanical strength of the femoral head. Moreover, the pores of bone tissue can be used as scaffolds to promote cell adhesion, proliferation, induction of osteoblasts, and integration and regeneration of bone tissue^45^. PRP is rich in various growth factors and plays an important role in tissue repair and mesenchymal stem cell proliferation and differentiation^18^. Experiments have substantiated that PRP can prevent ONFH by stimulating bone formation, vascularization, and retarding adipogenesis^46,47^. Clinically, PRP combined with autologous and allogeneic bone grafts has been associated with stronger bone healing ability and shorter healing time^48^.

RCTs included in the present study were not high-quality, and bias and possible methodological flaws weakened the strength of our findings to a certain extent. All included studies were from China and India, indicating that the application of PRP combined with core decompression and bone grafting for femur head necrosis is mainly used in Asia, which is suggestive of regional bias. Our findings should be interpreted with caution due to the presence of significant study limitations. Notably, the most significant risk of bias stems from the absence of blinding and intervention concealment, as only one study^20^ adopted double blinding. The primary outcome of relevant studies was based on the HHS, which is subjective. Moreover, besides obvious pathological changes (e.g. collapse and a significant increase in necrotic extent), even judgment of radiographic progression is subjective, emphasizing the importance of blinding. In fact, intervention concealment and implementation of blinding are not difficult to implement in these studies, requiring the collection of a predefined volume of venous blood and different clinicians in charge of the surgery and follow-up. The included studies did not describe the quality control of RPR and did not document the preparation process through description or literature citation. Various laboratory methods or commercial kits can produce PRP. Although no consensus has been reached on the optimal approach, double-step gradient centrifugation is the most common manual method^40^, involving two sequential centrifugation steps with different conditions of force, speed, and duration. During the first step, the whole blood sample was separated into three layers, yielding a lower layer containing red blood cells, a suspension (also called the acellular plasma layer sometimes, containing a lower concentration of platelets), and a middle buffy coat layer (containing leukocytes and platelets)^49^. After the second centrifugation step, the PRP collected from the upper suspension was called pure platelet-rich plasma (P-PRP), while the PRP produced from the buffy coat layer and the upper suspension was called leukocyte and platelet-rich plasma (L-PRP)^50^. The PRP prepared by single-step centrifugation was collected from the buffy coat layer. It has been confirmed that double centrifugation can yield higher concentrations of platelets and growth factors than single centrifugation^51^, exhibiting a stronger pro-bone regeneration potential in vivo^50^. Two studies^20,37^ used single-step centrifugation. Theoretically, PRP prepared by single centrifugation contains leukocytes. In this respect, one study^20^ used a leukocyte filter after centrifugation, and the other^37^ used single-step centrifugation.

In addition, another important source of bias is the difference in the centrifugation scheme (centrifugal force and duration). The rotational speed in the included studies was between 1500r and 3500r, and most studies did not specify the centrifugal radius. Accordingly, it was difficult to evaluate the heterogeneity of centrifugal force on conclusions. It has been shown that different platelet preparation procedures may differentially affect growth factor release and tissue repair^19,52^, which accounts for the lack of assertion on PRP’s clinical efficacy^40,53^. At the same duration, a higher centrifugal force can produce a higher platelet recovery rate, while an excessive centrifugal force and duration can also reduce the clinical efficacy of PRP for increased recovery of plasma and decreased shedding of platelets from sediments and aggregates^40^. In addition, high centrifugal force can damage platelets, cause premature platelet activation, and reduce platelet function^54^. The included studies lacked Processing Quantitative Standards (PQS) for PRP, and only one^37^ mentioned that the platelet count of the obtained PRP product was 1.5–2-fold that of peripheral blood. Indeed, platelet baseline criteria documentation in studies would facilitate further investigation of the therapeutic effects of different concentrations. Many studies have reported a positive correlation between the concentration of platelets in PRP and its ability to promote repair^40^. The platelet concentration in therapeutic PRP is reportedly three to fivefold^55^ or four to sixfold^42^ higher than the baseline platelet number in whole blood, although no consensus has been reached for the optimal platelet concentration in platelet derivatives. In terms of bone repair, *in vitro* studies have reported that 2.5-fold platelet concentration has better results for the proliferation and function of fibroblasts and osteoblasts^56^. However, it has been suggested that platelet concentrations four to fivefold higher than the normal value promote bone and soft tissue repair; at excessively high concentrations, the opposite effect would be achieved^57^. Indeed, the optimal platelet concentration for bone repair is affected by the environmental conditions since PRP contains growth factors that promote osteogenesis, angiogenesis, and tissue regeneration, and dense granules expressed in platelets release molecules such as serotonin, adenosine triphosphate, and polyphosphate that negatively affect bone healing^58^. Careful consideration is required for various variables during PRP preparation, and to some extent, commercial kits can meet these requirements. A standardized product, at the very least, mitigates operational bias, ensuring relatively stable PRP quality. The simplified operation not only saves time but also reduces the risk of sample contamination^59^. Two^32,33^ of the included studies used special kits, both of which were from the same Chinese manufacturer. However, the high costs of processing kits remain challenging^55^. Besides, the use of activators during surgery is also a concern. It is widely acknowledged that platelets are generally in a resting state and need to be activated before treatment^60^. Since centrifugation, tissue collagen, and trauma bleeding may cause platelet activation, anticoagulants are generally added during centrifugation to prevent early spontaneous activation and aggregation of platelets^55^. Activators such as thrombin and calcium chloride are used before use to induce a rapid and sufficient release of soluble factors^61^. Only two studies^20,37^ reported using calcium chloride to activate PRP, and the remaining studies did not mention activators, which may be attributed to surgical trauma itself having an activation effect.

The possible mechanism of PRP in the treatment of femur head necrosis may include the following aspects: (1) Diverse growth factors released by PRP, such as vascular endothelial growth factor, transforming growth factor-beta, platelet-derived growth factor, insulin-like growth factor, epidermal growth factor, fibroblast growth factor^62^, accelerate mesenchymal stem cell differentiation, promote vascular regeneration, and synthesize extracellular matrix, etc.^40^. Additionally, a variety of cytokines released by PRP exert a synergistic effect^63^.

(2) PRP also contains fibronectin and fibrin membranes, which provide the three-dimensional structure required for tissue repair and a scaffold for biological activities such as cell proliferation and migration^64,65^. (3) Exosomes in PRP are also thought to contribute to tissue repair and regeneration. Exosomes are carriers of biologically active proteins, mRNA, and noncoding RNA and play a crucial role in cell-cell and platelet-cell communication^66^. PRP-derived exosomes have been confirmed to inhibit chondrocyte apoptosis, promote chondrocyte proliferation and migration, inhibit the secretion of inflammatory factors, and promote the synthesis and secretion of extracellular matrix through various signaling pathways^67–69^. (4) PRP can promote healing of damaged tissue by regulating inflammation^40^. Chemokines and histamine released by activated PRP enhance the permeability of local arteries and local aggregation of immune cells, then necrotic tissue and pathogens are engulfed by immune cells^70^. At the same time, PRP contains a variety of negative regulators of inflammation, which also play an important role in inducing inflammatory cell apoptosis, reducing the inflammatory response and injury degree, and promoting cell proliferation^71,72^.

In addition to methodological and implementation limitations, the evaluation of results faced significant constraints. Indeed, the follow-up duration of included studies was insufficient since it is widely acknowledged that indicators of qualitative change in outcomes, such as collapse and treatment failure, should be followed for a longer period. The follow-up duration of most included studies was 6 or 12 months, and only two were longer than a year. Moreover, the evaluation of clinical efficacy depends on HHS, and some studies even graded the prognosis evaluation of cured, effective, acceptable, and ineffective according to the HHS. The good-to-excellent results parameter analyzed in this review was also graded based on HHS, which has many shortcomings, highlighting the change in postoperative pain and placing less emphasis on joint activity. It is widely believed that it is better to have a motionless and painless medullary joint than a mobile and painful one. On the other hand, the measurement of joint range of motion is often subjective, and excessive weight can cause poor repeatability of the scoring results. A pain-focused activity scale superimposed on the VAS pain score may significantly weaken the overall assessment of joint function. Although other modified HHSs have been designed, their utilization rate has been relatively low. These findings highlight the importance of the quest to identify a more unified and balanced evaluation scale for femur head necrosis. The studies included were all from Asia, and in addition to the fact that this combination therapy is more widely used in Asia, studies published in other languages (only studies published in English and Chinese were included) were not taken into account.

While the systematic review and synthesis were conducted with rigor, inherent limitations that cannot be avoided exist. Firstly, the evidence was not of high-quality due to clear methodological and implementation limitations in the current studies, significant heterogeneity, and a moderate risk of bias. Secondly, the long-term efficacy of PRP in the treatment of femur head necrosis was unclear because the included studies do not provide a longer follow-up. Finally, there was the potential for publication bias, and studies in non-English or non-Chinese languages might have been overlooked. Despite these limitations, our meta-analysis had prominent strengths. On the one hand, it was the first meta-analysis comparing the clinical efficacy between core decompression and bone grafting with and without PRP in the treatment of femur head necrosis. On the other hand, the inclusion and exclusion criteria were strictly controlled. Sources of bias in studies and heterogeneity of the conclusions were fully analyzed and explained.

## Conclusion

Core decompression and bone grafting combined with PRP demonstrate superior efficacy in terms of function, pain relief, and radiographic progression compared to procedures without PRP. However, further quality RCTs are needed to evaluate their effectiveness due to methodological and implementation limitations present in the existing evidence.

## Ethical approval and consent to participate

Ethical approval and patient consent are not relevant to this study since it is based on previously published studies.

## Sources of funding

This project was supported by the 2024 Shanghai Sports Science and Technology Project (24J020). It was also supported by the Budget Project of the Shanghai University of Traditional Chinese Medicine (2021LK095).

## Author contribution

B.Z. and B.L.: conceived this study and prepared the original draft; B.Z. and S.F.: designed this study, performed literature researches, and extracted data; B.Z., S.F., J.L., X.L., and B.L.: contributed to the revision of the manuscript. Final approval of the manuscript has been obtained from all authors.

## Conflicts of interests disclosure

The authors declare that they have no competing interests.

## Research registration unique identifying number (UIN)

1. Name of the registry: PROSPERO International prospective register of systematic review.
2. Unique identifying number or registration ID: CRD42022361007.
3. Hyperlink to your specific registration (must be publicly accessible and will be checked): https://www.crd.york.ac.uk/PROSPERO/display_record.php?RecordID=361007

## Guarantor

Shengyi Feng and Bo Li.

## Data availability statement

This article and its supplementary information files contain all data generated during this study.

## Provenance and peer review

Not commissioned, externally peer-reviewed.

## Supplementary Material

SUPPLEMENTARY MATERIAL
